# Clinicopathological Significance of Syndecan-1 in Cholangiocarcinoma: A Study Based on Immunohistochemistry and Public Sequencing Data

**DOI:** 10.3390/jcm10132745

**Published:** 2021-06-22

**Authors:** Tiemo S. Gerber, Fabian Bartsch, Daniel C. Wagner, Mario Schindeldecker, Lisa-Katharina Heuft, Wilfried Roth, Hauke Lang, Beate K. Straub

**Affiliations:** 1 Institute of Pathology, University Medical Center of the Johannes Gutenberg-University Mainz, 55131 Mainz, Germany; tiemo.gerber@unimedizin-mainz.de (T.S.G.); Daniel-Christoph.Wagner@unimedizin-mainz.de (D.C.W.); mario.schindeldecker@unimedizin-mainz.de (M.S.); wilfried.roth@unimedizin-mainz.de (W.R.); 2 Department of General, Visceral and Transplant Surgery, University Medical Center of the Johannes Gutenberg-University Mainz, 55131 Mainz, Germany; fabian.bartsch@unimedizin-mainz.de (F.B.); lisa-katharina.heuft@unimedizin-mainz.de (L.-K.H.); Hauke.Lang@unimedizin-mainz.de (H.L.); 3 Tissue Biobank, University Medical Center of the Johannes Gutenberg-University Mainz, 55131 Mainz, Germany

**Keywords:** cholangiocarcinoma, syndecan-1, biomarker, *SDC1*

## Abstract

Background: Syndecan-1 (CD138; *SDC1*) is a heparan sulfate proteoglycan that has been attributed a key role in cancer progression in ductal adenocarcinoma of the pancreas. We therefore aimed to investigate the role of syndecan-1 in cholangiocarcinoma. Methods: We analyzed syndecan-1 expression in a large, clinicopathologically well-characterized collective of 154 intrahepatic cholangiocarcinoma, 221 extrahepatic cholangiocarcinomas, and 95 gallbladder carcinomas as well as respective normal tissues and precursor lesions by immunohistochemistry with digital image analysis and correlated with recurrence-free survival and prognostic markers. Furthermore, we conducted an analysis of cancer genes in the cholangiocarcinoma cohort of The Cancer Genome Atlas (TCGA). Results: During cholangiocarcinogenesis, syndecan-1-expression decreased when compared to normal bile ducts and biliary intraepithelial neoplasia; however, syndecan-1 levels were found to be elevated in lymph node metastases. In the TCGA cohort, high mRNA *SDC1* levels were associated with poor prognosis in intrahepatic cholangiocarcinoma. However, in our large cohort, the immunohistochemical syndecan-1 expression did not significantly correlate with recurrence-free survival. Conclusions: Syndecan-1 was found to be downregulated during cholangiocarcinogenesis, yet we could not show significant effects on prognosis on protein level. Further analyses are needed to further depict its specific role.

## 1. Introduction

Syndecans are a family of cell surface heparan proteoglycans. Syndecan-1 (CD138, encoded by the *SDC1* gene [1]) is a transmembrane protein with an intra- and extracellular domain. It is expressed on the basolateral surface of epithelial cells, binds with its extracellular domain to extracellular matrix components, and associates with its intracellular domain to the actin cytoskeleton. Syndecan-1 plays a key role in the modulation of cancer cell proliferation and invasion, inflammation, and matrix remodeling. In the liver, syndecan-1 is expressed on the sinusoidal and intercellular surface of hepatocytes and the basolateral surface of cholangiocytes of bile ducts, irrespective of size. Immunohistochemical expression is increased in specimens with liver cirrhosis and chronic cholestatic disease [2,3,4].

CD138 immunohistochemistry is currently widely used in routine histopathology to highlight plasma cells in different immunologic diseases as well as for the diagnosis of plasma cell myeloma. Anti-CD138 drugs are currently being evaluated in clinical trials for this disease [5].

Syndecan-1 was recently uncovered to be upregulated at the cell surface by *KRAS* in pancreatic ductal adenocarcinoma (PDAC). *KRAS* mutations are the most common and earliest alteration in cancer development in PDAC, which is present in more than 90% of cases. Syndecan-1 is supposed to mediate macropinocytosis at the cell surface, which is involved in cancer growth and progression. In addition, macropinocytosis is associated with necrocytosis, a drug resistance mechanism for standard chemotherapeutic therapies, such as gemcitabine, 5-fluorouracil, and doxorubicin [6,7]. *KRAS* mutations, macrocytosis, and drug resistance are closely related factors [8,9,10]. Therefore, these findings are attracting considerable interest to biomarkers with a prognostic relevant association to macropinocytosis. For syndecan-1, a mechanistic link to *KRAS* signaling and macropinocytosis has recently been uncovered. This critical role of *SDC1* has led to the suggestion of therapeutic interventions with antibody targeting [7,11].

PDAC and carcinomas of the biliary tract stem from embryonically related cell types, and therefore might share similarities in tumor biology. Cholangiocarcinomas are a rare and heterogeneous group of malignant tumors with globally rising incidence. It is the second most common primary hepatic malignancy behind hepatocellular carcinoma. In the past decade, the prognosis of cholangiocarcinoma patients has not improved substantially [11]. Intrahepatic cholangiocarcinomas are aggressive malignancies with poor overall survival and a high probability of recurrence, even with negative tumor margin resection [12]. We aimed to elaborate whether syndecan-1 expression correlates to survival in cholangiocarcinoma and thereby parallels its function in PDAC.

## 2. Materials and Methods

Paraffin-embedded tissue samples from 470 patients with cholangiocarcinoma were provided by and in accordance with the regulations of the Tissue Biobank of the University Medical Center Mainz after approval by the local ethics committee of Rhineland-Palatinate. In our series, we included intra- and extrahepatic cholangiocarcinoma (iCCA and eCCA) as well as gallbladder carcinoma (GBC, *n* = 95), diagnosed at the Institute of Pathology, University Medical Center Mainz, between the years 2006 and 2020. Tissue samples were obtained from surgical specimens, and larger tissue samples were obtained from irresectable cases. Intrahepatic cholangiocarcinoma (*n* = 154) were reviewed and classified into small and large duct types according to the WHO classification of tumors of the digestive system (fifth edition, 2019). Depending on its location, ECCA were subdivided into perihilar (PHCC, *n* = 162) and distal (dCCA, *n* = 59) cholangiocarcinoma. PHCC were defined as primarily located in the hilus region in the common hepatic duct and possibly extending to the right or left hepatic duct with periductal growth. Small duct-type iCCA were defined as typically peripherally localized carcinomas with small tubular growth, low columnar tumor cells, without mucin secretion, and typically without perineural invasion, whereas iCCA, large duct type, were by definition typically centrally/periductally located carcinomas with the formation of large ducts and mucin production [13]. In addition, we also included biliary intraepithelial neoplasia (BilIn) and normal tissue (small, large and perihilar bile ducts, and gallbladder epithelium). Clinical follow-up and survival data were available in most cases (Table 1). Tissue microarray (TMA) blocks were generated with primary cancer, corresponding normal tissue, precursor lesions, and metastases. Subsequently, TMA images were digitalized by a whole slide scanner at 400×, with a pixel size of 0.2278 × 0.2278 µm (Nanozoomer, Hamamatsu Photonics, Hamamatsu, Japan) and further analyzed. 

**Figure 1:**
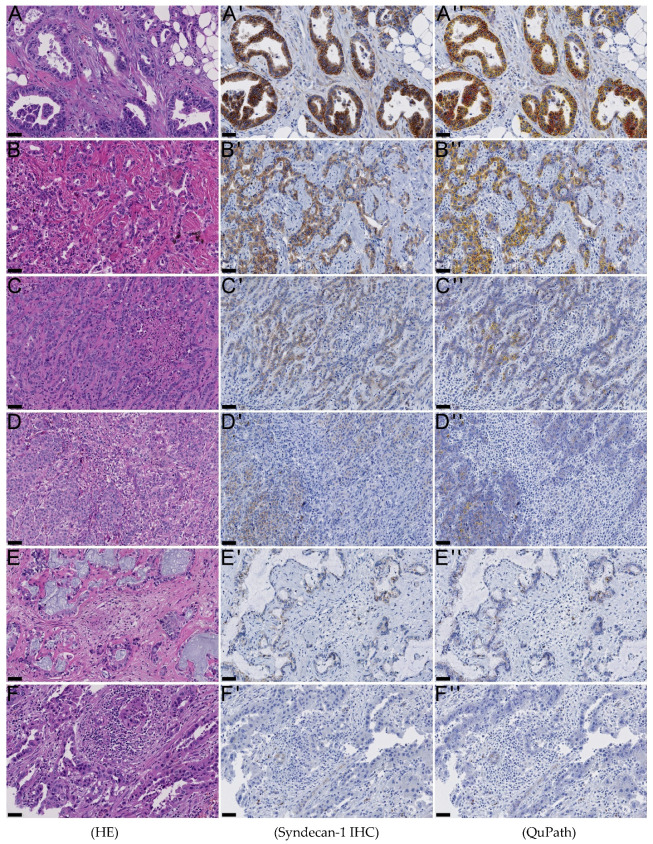
Scoring of syndecan-1 immunohistochemistry: *Manual score*: Score 3.0 (**A’**), 2.5 (**B’**), 2.0 (**C’**), 1.5 (**D’**), 1.0 (**E’**), 0.5 (**F’**). *QuPath H-score* 90.00 (**A’’**), 52.62 (**B’’**), 1.84 (**C’’**), 1.02 (**D’’**), 0.99 (**E’’**), 0.05 (**F’’**). Intrahepatic cholangiocarcinoma of small (**C**,**D**) and large (**A**,**B**,**E**,**F**) duct type are depicted. Classification of tumor staining intensity: red circumference 3+, orange 2+, yellow 1+, blue 0; stroma in white (**A’’**–**F’’**). Black bars: each 50 µm.

Immunohistochemistry (IHC) was performed on formalin-fixed, paraffin-embedded sections, cut at maximum 4 µm thickness. Syndecan-1 expression was detected using a mouse anti-human monoclonal antibody (CD138 Clone MI15, Dako, Germany) in a dilution of 1:1000, according to the manufacturers’ recommendations. IHC stains were manually evaluated on each TMA core by scoring intensity between 0 and 3 (Figure 1) [14]. To improve accuracy, we scored in 0.5 increments. Complete circumferential membrane staining was defined to be at least a score of 1.5, the actual score depending on intensity. Manual scoring aimed for qualitative assessment of tumor cells, counting even single tumor cells. For patients with more than one TMA core of the same tumor taken for internal assessment of tumor heterogeneity, we calculated the mean value. 

Quantitative assessment of syndecan-1 expression levels was performed using QuPath, an open-source bioimage analysis software, version 0.2.3 [15]. TMAs cores were de-arrayed. Stain vectors and background vectors were individually set in each slide and further analyzed using the “cell detection” algorithm in QuPath. Cellular chromogen 3,3’-diaminobenzidine-tetrahydrochloride-dihydrate mean levels were classified in four categories using empirical threshold scores (0–0.21 for score 0, >0.21 for 1+, >0.45 for 2+, and >0.7 for 3+; see Figure 1). These levels provided a relatively low threshold for the discrimination of negative stains (score 0) and low positivity (score 1+), and a relatively high threshold for the highest achievable score (score 3+). Tumor cells were annotated using a detection classifier. Due to intertumoral heterogeneity, we used custom-tailored classifiers on a case-to-case basis to ensure proper separation of tumor and non-tumor tissue. This entailed the application of the random trees classifier to train QuPath interactively to distinguish tumor cells from stromal cells. The H-score was calculated according to established practice. It is calculated from the extent and intensity of staining, giving a score range of 0 to 300 [16]. In addition, to further elucidate the relationship between syndecan-1, *KRAS*, and patient prognosis, we analyzed the publicly available cBioPortal for Cancer Genomics data for cholangiocarcinoma data from the Cancer Genome Atlas (TCGA) and non TCGA data [17,18,19]. 

Using the syndecan-1 H-scores from our cohort and the *SDC1* mRNA data from the TCGA cohort, both optimal cut-off values were calculated using the Charité Cutoff Finder [20]. Survival analyses were plotted by the Kaplan–Meier model and compared by the log-rank test. For the survival analysis of our cohort, we used only primary iCCA, and we excluded cases with irresectability and recurrent tumors. The database patient/TMA-core assignment was conducted using the MS-Access 2016 to achieve referential integrity. We calculated the Spearman rank-order correlation coefficient for ordinal data and Pearson correlation coefficient for metric data using SPSS v27.0.1.0 (two-sided). To compare the means of two groups, we used Student’s *t*-test. A *p*-value < 0.05 was considered statistically significant.

## 3. Results

### 3.1. Syndecan-1 Expression Was Downregulated during Cholangiocarcinogenesis

**Figure 2:**
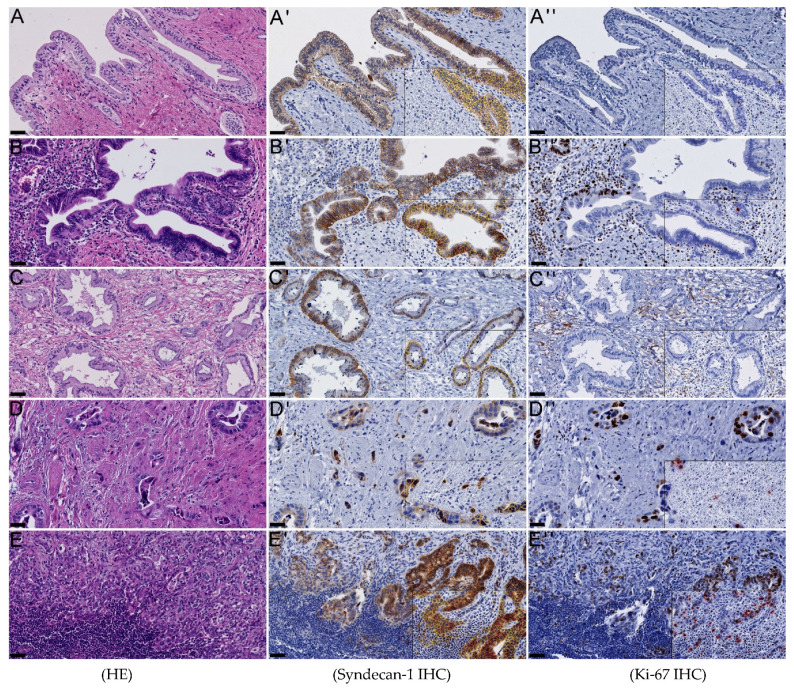
Syndecan-1 expression decreased in cholangiocarcinoma: In normal bile ducts (**A**), biliary intraepithelial neoplasia (**B**), and lymph node metastases (**E**), syndecan-1 staining was higher than in well- (**C**) and poorly differentiated (**D**) cholangiocarcinoma. (**A**) Perihilar bile duct, (**B**) low-grade BilIn, (**C**–**E**) PHCC. Syndecan-1 H-score: 79.81 (**A**), 81.22 (**B**), 45.20 (**C**), 34.70 (**D**), 106.31 (**E**). Ki-67: 0.06% (**A**), 9.07% (**B**), 0.98% (**C**), 41.67 (**D**), 32.51 (**E**). The overlay depicting QuPath-analysis is shown each on the bottom right (**A’**–**E’**, **A’’**–**E’’**). Classification of tumor staining intensity: red circumference 3+, orange 2+, yellow 1+, blue 0; stroma in white (**A’**–**E’**); red circumference positive, blue negative; stroma in white (**A’’**–**E’’**). Black bars: each 50 µm.

To investigate syndecan-1 in cholangiocarcinoma, we performed immunohistochemical analyses using our large cohort of over 470 iCCA, PHCC, dCCA, and GBC. Syndecan-1 was uniformly expressed cytoplasmically and at the cell membranes of hepatocytes and occasionally of bile ducts, as well as in singular plasma cells in normal liver. According to the literature, carcinomas have been attributed a “honeycomb” staining pattern as well heterogenous staining intensity of cell membranes and cytoplasm [4,21,22]. At least weak (intensity score ≥ 0.5) syndecan-1 expression was detected in 81.42% of iCCA, 62.39% of PHCC, 41.18% of eCCA, 44.86% of GBC, 85.94% of BilIn, 76.19% of small bile ducts, 78.38% of large and perihilar bile ducts, and 28.57% of gallbladder epithelium. To our knowledge, only one study has analyzed immunohistochemical syndecan-1 expression in cholangiocarcinoma, revealing a positivity in 39.1%, without subclassification [5]. Overall, BilIn (*n* = 155) showed a comparable mean syndecan-1 H-score (94.36 ± 65.42) to large and perihilar bile ducts (*n* = 91; 91.46 ± 67.53), (*p* = 0.741). The mean H-score of iCCA, dCCA, PHCC, and GBC had a significantly lower mean syndecan-1 H-score than normal bile ducts (*p* < 0.001), arguing towards a significant downregulation of syndecan-1 during cholangiocarcinogenesis (see Figure 2). Lymph node metastases (*n* = 126; 52.53 ± 50.11), however, showed a significantly higher mean value than primary carcinomas (*p* = 0.034). The mean intensity score in normal bile ducts was 1.63 ± 0.86, in BilIn 2.2 ± 0.73, in lymph node metastasis 1.61 ± 0.88, while primary carcinomas showed a mean score of 1.41 ± 0.81. On the contrary, according to Yao et al. (2020), in PDAC and pancreatic intraepithelial neoplasia, immunohistochemistry showed a higher syndecan-1 intensity than in normal tissue [7].

### 3.2. Low Syndecan-1 mRNA Levels Were Associated with Better Survival

**Figure 3:**
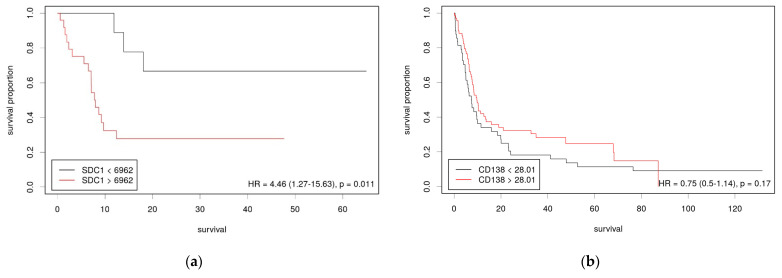
Kaplan–Meier curves for disease-free survival of cholangiocarcinoma (**a**) and recurrence-free survival of intrahepatic cholangiocarcinoma (**b**). Patients stratified by (**a**) *SDC1* mRNA expression (TCGA cohort; *n* = 36) and (**b**) syndecan-1 immunohistochemical expression level (own cohort; *n* = 122). *x*-axis: time in months (**a**,**b**).

To comprehensively investigate syndecan-1 concerning patient prognosis, we conducted survival analysis (Figure 3). In our cohort, 122 patients met the inclusion criteria (see Table 1). Low mRNA levels of syndecan-1 (gene symbol *SDC1*) were significantly associated with a better prognosis (cutoff value 6962, area under the curve (AUC) = 0.64; see Figure 3a). In the TCGA cohort, two patients showed a missense mutation of KRAS. Both were in the high *SDC1* mRNA group. Immunohistochemical expression of syndecan-1, as evaluated by H-scores, could not separate any prognostically relevant groups (see Figure 3b). The best cutoff value in our cohort was the H-score of 28.01 (AUC = 0.55).

Additionally, in iCCA, we performed a correlation analysis between the syndecan-1 H-score and the residual tumor classification, the lymph node status, the iCCA subclassification (small and large duct type), and the Ki-67 proliferation rate. As expected, the proliferation rate correlated with histological tumor grade [23]. No statistically significant association between the H-score and the prognostically important marker proliferation rate, residual tumor status, lymph node status, and histological subtype analysis was detected (Ki-67: ρ = 0.08 (*p* = 0.925), R-status: ρ = 0.062 (*p* = 0.484), iCCA subclassification ρ = 0.002 (*p* = 0.985). In primary carcinomas, syndecan-1 did not correlate with tumor size ρ = 0.14 (*p* = 0.835), grading ρ = 0.063 (*p* = 0.187), or GPT-value (ρ = 0.49 (*p* = 0.714)). However, syndecan-1 correlated weakly with age ρ = 0.138 (*p* = 0.003).

### 3.3. Molecular Analysis

In the overall cBioPortal data, *KRAS* was mutated in 122 of 805 cholangiocarcinoma cases (15.2%; not shown). Most *KRAS* mutations were G12D (45), G12V (39), and G12C (7). In the TCGA cohort, two *KRAS* mutations were detected. *SDC1* mRNA levels were not overtly associated with *KRAS* mutational status (Figure S1). In all cBio Portal cholangiocarcinomas with known progression-free survival, *KRAS* mutations were associated with a poor prognosis (*p* < 0.001; see Figure S2).

## 4. Discussion

This is the first study to comprehensively study syndecan-1 in cholangiocarcinoma to unravel its possible role as a therapeutic agent, as already shown for PDAC. We could demonstrate positivity in most iCCA, dCCA, GBC, PHCC, BilIn, and associated normal tissue. In our study cohort of iCCA, the immunohistochemical expression did not correlate with recurrence-free survival. Only one other study, conducted by Harada and coauthors (2003), had previously addressed the question as to whether syndecan-1 is associated with patient prognosis in cholangiocarcinoma. This study demonstrated an association between reduced immunohistochemical expression of syndecan-1 and reduced recurrence-free survival [4]. However, this study only included 33 cases, of which 13 (39%) were poorly differentiated. Poor differentiation significantly correlated with syndecan-1 expression. We assume this served as a confounder, explaining the different results. In addition, the concept of subclassification of large and small duct type cholangiocarcinomas arose more than a decade after the aforementioned study, which may have impacted the different prognosis as well [13,24]. 

mRNA levels could differentiate two different prognostically significant groups in the TCGA cohort. However, this was a small group (*n* = 36) compared to our large cohort (*n* = 122). mRNA levels may only be comparable to a limited extent to protein levels, as the extent to which the mRNA is translated to a protein may vary. In addition, high *SDC1* mRNA levels in whole tissue lysates may originate from tumor-infiltrating plasma cells and not only carcinoma cells. Immunohistochemistry, on the other hand, is direct, though semiquantitative measurement of the protein of interest and evaluation concentrates on tumor cells only. Therefore, immunohistochemistry is widely used in routine pathology as a derivate marker, e.g., for the analysis of Her2 expression in breast cancer. 

Syndecan-1 is a membrane-bound protein. However, regulated nuclear translocation has been described via association with tubulin in the mitotic spindle [25]. These findings suggest an association with cell proliferation and tumor aggressiveness. However, in our study, there was no positive correlation with the Ki-67 proliferation index. 

Elevated syndecan-1 expression had been demonstrated in *KRAS-*driven PDAC [7]. In our series, syndecan-1 staining intensity was significantly lower in cholangiocarcinoma when compared to normal bile ducts. There was no significant difference between normal bile ducts and biliary intraepithelial neoplasia. In intrahepatic cholangiocarcinoma, syndecan-1 H-score had no significant influence on recurrence-free survival, although *KRAS-*mutation status had an influence on recurrence-free survival in cholangiocarcinoma of the cBioPortal cholangiocarcinoma cohort. This discrepancy in syndecan-1 immunohistochemistry may have multiple reasons. First and foremost, *KRAS* mutations are much less common in cholangiocarcinoma (15.2%) when compared to PDAC (more than 90%). Due to *KRAS*-dependent changes, syndecan-1 is upregulated in PDAC, as stated by Yao et al. [7]. For this reason, to reliably compare cholangiocarcinoma and PDAC, two subgroups may be needed to further depict an independent role of syndecan-1, namely, *KRAS-*mutated and *KRAS*-wildtype tumors. Second, PDAC and cholangiocarcinoma are embryonal related but show distinct tumor biology. This may result in different tumor evolution and cell signaling. In comparison to our molecular analysis, Robertson et al. found 7.4% *KRAS* mutations (G12D) in a cohort of 54 intrahepatic cholangiocarcinomas, which were associated with a worse long-term overall survival [26]. In cholangiocarcinoma, the frequency of *KRAS* mutations is dependent on the subtype. In a recent study, Goeppert et al. demonstrated 23.3% *KRAS* alterations in iCCA, 40.6% in PHCC, 16.7% in dCCA, and 24.4% in GBC. Patients with tumors harboring *KRAS* mutations showed a significantly poorer survival [27]. These concordant findings underline the importance of further studies regarding the role of syndecan-1 in cholangiocarcinoma.

Our study has several limitations. First, syndecan-1 expression on tumor cells using immunohistochemistry is on a continuous spectrum, whereas in our evaluation, categorical and metrical variables need to be defined. To compensate for this, we manually scored each TMA core, counting even single cells, and, additionally, for quantification, we calculated the H-score. Second, in this study, we did not perform molecular analyses to determine whether *SDC1* mRNA levels may correlate with immunohistochemical expression. Third, we did not compare different chemotherapy approaches for intrahepatic cholangiocarcinoma with syndecan-1 expression, although there may be an association. However, in our study using predominantly operation specimens of patients undergoing resection with curative intent, there had not been a sufficient number of patients that underwent prior chemotherapy treatment, especially considering the many subgroups needed for this analysis. Recent and future advances in therapies may increase the proportions of patients with neoadjuvant treatment [12,28]. Fourth, we investigated cholangiocarcinoma TMAs instead of whole slides. Although TMAs are a generally accepted method, naturally, immunohistochemistry of whole slides may have led to more accurate data. To compensate for this, in our series, we used more than 2100 2-mm measuring TMA cores, resulting in multiple scores per case. 

To conclude, we could not show an impact of syndecan-1 on patient prognosis in cholangiocarcinoma, at least none that could be shown immunohistochemically. It remains unclear as to whether *SDC1* mRNA levels may reliably predict prognosis. Given the importance of syndecan-1 in PDAC, this is rather surprising and should warrant further investigation. Further experimental investigations are therefore needed to clarify whether syndecan-1 may predict chemotherapy response in cholangiocarcinoma and whether macropinocytosis may be involved in cholangiocarcinoma progression.

## Figures and Tables

**Table 1:** Patient characteristics.

	iCCA *n* = 154	dCCA *n* = 59	PHCC *n* = 162	GBC *n* = 95
Age ^†^	64.02 ± 10.78	69.04 ± 9.01	66.94 ± 11.01	67.17 ± 11.92
Male	59%	71%	62%	35%
Female	41%	29%	38%	65%
**Syndecan-1**				
Intensity ^†^	1.72 ± 0.77	1.30 ± 0.89	1.46 ± 0.82	1.17 ± 0.84
H-score ^†^	47.5 ± 48.22	38.28 ± 44.59	44.16 ± 42.64	39.67 ± 47.67
**Proliferation** ^‡^	17.75 ± 15.83	21.91 ± 16.46	18.39 ± 15.92	23.94 ± 17.09
pTX	15	0	12	11
pT1	75	7	8	3
pT2	41	26	122	30
pT3	18	25	18	47
pT4	5	1	2	4
pNX	51	0	32	31
pN0	70	31	87	31
pN1/2	33	28	43	33
G1	5	1	5	3
G2	117	37	104	43
G3	32	19	53	47
G4	0	2	0	2
L0	135	42	141	65
L1	19	17	21	30
V0	128	50	145	81
V1	26	9	17	14
Pn0	125	16	54	55
Pn1	29	43	108	40
R0	111	49	117	48
RX	18	5	19	14
R1	25	5	26	33

^†^ mean value ± standard deviation. ^‡^ measured in % immunoreactive tumor cells for Ki-67. G1: well-differentiated; G2: moderately differentiated; G3: poorly differentiated; G4: undifferentiated. L0: No lymphangio invasion. L1: lymphangio invasion. V0: no vascular invasion; V1: vascular invasion. Pn0: no perineural invasion; Pn1: perineural invasion. R0: no residual tumor; R1: microscopic residual tumor; RX: residual tumor unknown.

## Data Availability

Publicly available datasets were analyzed in this study. The results shown here are in part based upon data generated by the cBioPortal for Cancer Genomics data for cholangiocarcinoma. The data that support the findings of this study are available on request from the corresponding author. The data are not publicly available due to containing information that could compromise the privacy of research participants.

## Supplementary Materials

The following are available online at https://www.mdpi.com/article/10.3390/jcm10132745/s1, Figure S1: TCGA mRNA analysis of cholangiocarcinoma, Figure S2: *KRAS* mutations in cholangiocarcinoma.
