# Supplementary material for: Clinicopathological Significance of Syndecan-1 in Cholangiocarcinoma: A Study Based on Immunohistochemistry and Public Sequencing Data
Source: J Clin Med. 2021 Jun 22;10(13):2745. doi: 10.3390/jcm10132745 (PMC8269152; doi:10.3390/jcm10132745)
Supplement: Supplementary file 1 [file jcm-10-02745-s001.zip › jcm-1241416-supplementary.pdf]

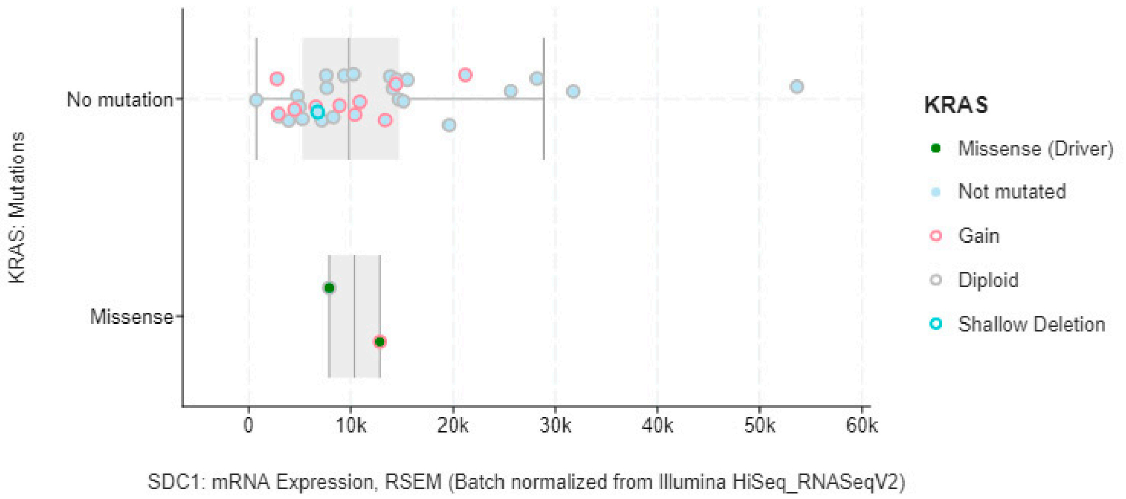

Figure S1. TCGA mRNA analysis of cholangiocarcinoma.

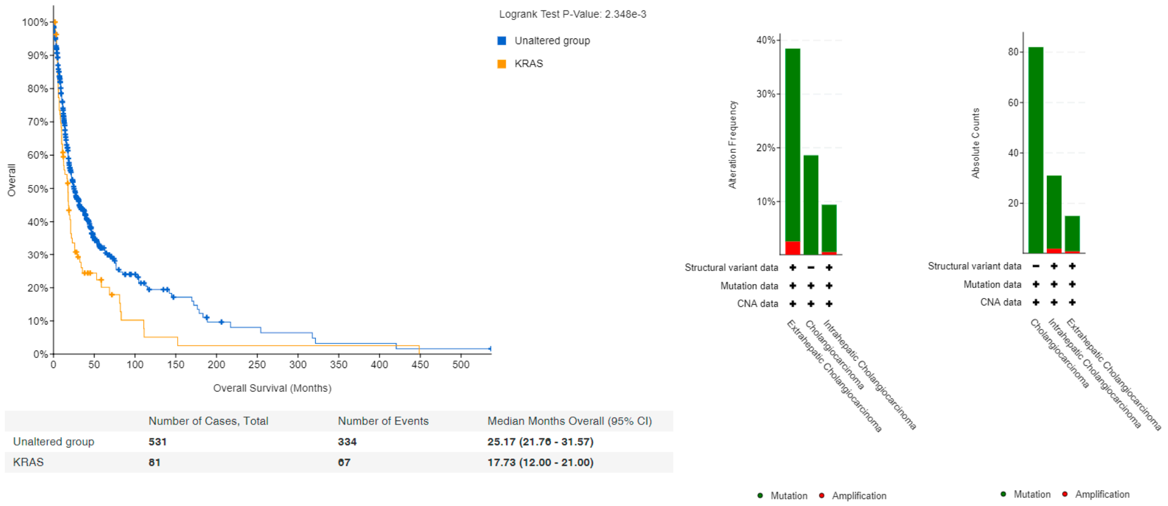

Figure S2. KRAS mutations in cholangiocarcinoma.
